# Optical coherence tomography angiography for the diagnosis of choroidal neovascularization in age-related macular degeneration: a systematic review

**DOI:** 10.31744/einstein_journal/2025RW1521

**Published:** 2025-11-07

**Authors:** Tarciana de Souza Soares, Amanda dos Santos Cristino, Analmiria de França Silva, Leticia Ribeiro dos Santos

**Affiliations:** 1 Universidade Federal de São Paulo Escola Paulista de Medicina Department of Ophthalmology and Visual Sciences São Paulo SP Brazil Department of Ophthalmology and Visual Sciences, Escola Paulista de Medicina, Universidade Federal de São Paulo, São Paulo, SP, Brazil.

**Keywords:** Macular degeneration, Optical coherence tomography, Angiography, Wet macular degeneration, Choroidal neovascularization

## Abstract

**Objective:**

To evaluate the diagnostic accuracy of optical coherence tomography angiography for the identification of choroidal neovascularization in age-related macular degeneration.

**Methods:**

This systematic review followed the Preferred Reporting Items for Systematic Reviews and Meta-Analyses (PRISMA) 2020 recommendations. A literature search of the PubMed/MEDLINE, Scientific Electronic Library Online (SciELO), EMBASE, Cochrane Library, CAPES Periodicals, and LILACS scientific databases was conducted to identify relevant full-text articles published in English and Portuguese from January 2012 to January 2025. To comprehensively evaluate the effectiveness and the performance of optical coherence tomography angiography in terms of sensitivity and specificity, fundus fluorescein angiography (the gold standard for detecting choroidal neovascularization) was used as the comparator. The Quality Assessment of Diagnostic Accuracy Studies (QUADAS) 2 tool was used to assess methodological quality and risk of bias.

**Results:**

Eleven articles were included in the systematic review. The patients’ mean age ranged from 58.5 to 79.7 years. The sensitivity ranged from 50.0% to 94.0%, and the specificity ranged from 67.6% to 100.0%. The risk of bias was low, and the methodological quality of the studies was good, suggesting that optical coherence tomography angiography holds promise for the diagnosis of choroidal neovascularization.

**Conclusion:**

Optical coherence tomography angiography exhibited high sensitivity and specificity, demonstrating its potential for the detection of choroidal neovascularization in patients with age-related macular degeneration. However, current scientific evidence suggests that optical coherence tomography angiography is not superior to fundus fluorescein angiography and should not be used as a substitute. Extended and affordable protocols to analyze different subtypes of choroidal neovascularization and the performance of optical coherence tomography angiography devices should be evaluated.

**Prospero database registration:**

CRD420251046669.

## INTRODUCTION

Age-related macular degeneration (AMD) is a degenerative disease of the retina that affects patients aged 50 years or older in its early, intermediate, or late stages and is one of the main causes of irreversible blindness.^(1–3)^ Age-related macular degeneration affects approximately 200 million people worldwide, and its prevalence is expected to increase over time as a consequence of population aging.^(4)^

The late stage of the disease implies the presence of either atrophic AMD (non-neovascular/"dry") or exudative AMD (neovascular/"wet"). Neovascular age-related macular degeneration (nAMD) accounts for 90% of all cases of AMD-related blindness caused by macular neovascularization (MNV), which involves the growth of abnormal blood vessels in the macular region induced by the increased expression of hypoxia-induced vascular endothelial growth factor A (VEGF-A).^(5)^ The CONAN Study Group criteria have been used to define the specific nomenclature of MNV based on multimodal imaging.^(2)^ The present systematic review included the diagnosis of choroidal neovascularization (CNV), and the spectrum considered the anatomical location of the lesion according to the following subtypes: type 1; type 2; mixed types 1 and 2; and type 3. Ensuring an early diagnosis of CNV can contribute to improved AMD management, increasing both the likelihood of beneficial outcomes from the treatment regimen and a better prognosis associated with central visual loss.

Fundus fluorescein angiography (FFA) has long been considered the gold standard for the diagnosis, classification, and ophthalmological management of this disease; however, optical coherence tomography angiography (OCTA) has recently emerged as a valuable non-invasive tool for studying blood flow in retinal structures and choroid microcirculation without requiring dye injection.^(6)^ OCTA has helped elucidate the pathophysiological mechanisms underlying AMD, and the refinement of the technique allows for a personalized therapeutic approach.^(7)^

Optical coherence tomography angiography assesses both functional (blood flow) and morphological (fluid accumulation) features of the CNV area comprising the choroidal neovascular network, characterized by its shape, branching, anastomoses, the type of vessel termini, and the presence of a choriocapillaris dark halo.^(8,9)^ Optical coherence tomography angiography can be used for the monitoring of lesion growth during follow-up, which can facilitate AMD therapy, as well as for the identification of CNV as a useful biomarker of disease progression and activity. Previous systematic reviews have reported inconsistent sensitivities and specificities across studies involving OCTA, which may have been influenced by factors such as the methodology and equipment used.^(10,11)^ Optical coherence tomography angiography has been demonstrated to be comparable to FFA and useful in facilitating the identification of CNV in AMD.^(12)^

## OBJECTIVE

The aim of this systematic review was to evaluate the diagnostic performance (sensitivity and specificity) of optical coherence tomography angiography in the detection of CNV and the diagnosis of AMD, with FFA used as the comparative method of evaluation.

## METHODS

This systematic review was conducted in accordance with the Preferred Reporting Items for Systematic Reviews and Meta-Analyses (PRISMA) 2020 statement.^(13)^ A literature search of the PubMed/MEDLINE, Scientific Electronic Library Online (SciELO), Excerpta Medica Database (Embase), Cochrane Library, CAPES Periodicals, and LILACS scientific databases was conducted to identify relevant full-text Portuguese- and English-language articles published from January 2012 through January 2025.

### Definition of the research question

Studies were selected to address the pre-specified research question, established using the population, intervention, comparator, and outcome (PICO) framework (Table 1); this enabled the selection of search terms (descriptors) and defined the logical structure for searching studies that evaluated the diagnostic performance of OCTA compared to that of the gold standard FFA for detecting CNV in AMD, with the primary outcomes being the sensitivity and specificity.

**Table 1:** PICO framework-based strategy for conducting database searches

Parameter	Definition	Study selection criteria
Population	Population included in the study	Patients with age-related macular degeneration
Intervention	Investigated intervention	Optical coherence tomography angiography
Comparator	The gold standard intervention most widely used	Fundus fluorescein angiography
Outcomes	The set of results selected to answer the clinical question	Diagnostic accuracy measures (sensitivity and specificity)

### Study eligibility criteria

The eligibility criteria, based on the PICO framework, included the following types of studies: diagnostic tests, cross-sectional studies, case-control studies, case series, and cohort studies. This systematic review excluded case reports, letters, editorials, bibliographic reviews, experimental studies involving animal models, textbooks (gray literature), manuals, and articles without full-text availability.

### Search strategy

The search strategies used Medical Subject Headings (MeSH), DECS, and Emtree descriptors with Boolean operators (delimiters) or connecting terms (AND, OR, and NOT) (Table 1S, Supplementary Material).

### Data collection and management

The Rayyan platform was used for the management and selection of the studies included in this systematic review.^(14)^ Three reviewers independently performed the bibliographic search using the pre-determined search strategies, screened titles and abstracts, reviewed the full-text versions of the articles, and extracted the data. Discrepancies were resolved by a fourth reviewer who compared the results against the inclusion criteria.

### Risk of bias assessment

The risk of bias was evaluated using the Quality Assessment of Diagnostic Accuracy Studies 2 (QUADAS-2) tool^(15)^ based on the following four separate domains: (i) patient selection; (ii) the index test (*i.e*., the diagnostic technique investigated in the study); (iii) the reference standard; and (iv) the patient flow and timing. Each domain was evaluated based on 14 questions, with the possible responses being "yes", "no", or "unclear"; "yes" and "no" responses related to low and high risks of bias, respectively. Collectively, depending on the responses, the risk of bias was classified as "low" (in green), "high" (in red), and "unclear" (in yellow) for the diagnostic applicability. The "unclear" category was assigned when a study reported insufficient data to permit a judgment.

Fulfillment of seven to nine criteria ("yes" answers) were considered to be the thresholds for considering the quality of each study to be "fair to good" and "good", respectively.

## RESULTS

**Figure 1:**
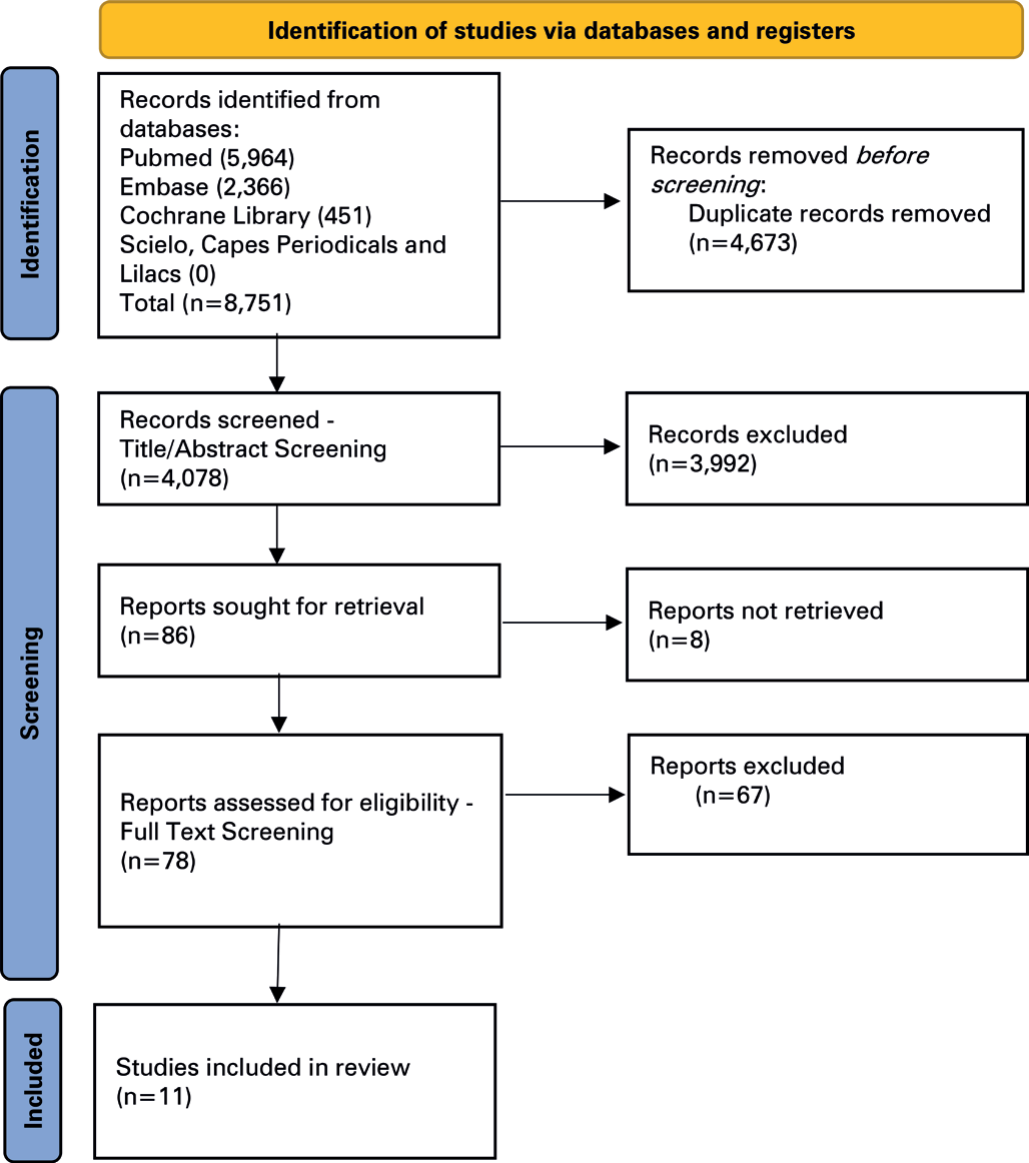
PRISMA flow chart of the study selection process

The literature search yielded 8,751 English-language articles, none of which were indexed in the SciELO, LILACS, and CAPES Periodicals databases. After removing duplicates (n=4,673), 4,078 articles were screened based on their titles and abstracts, yielding 78 articles eligible for full-text review. Eleven studies met the eligibility criteria and were ultimately included in the analysis. A Preferred Reporting Items for Systematic Reviews and Meta-Analyses (PRISMA) flowchart of the study selection process is shown in figure 1.

### Study characteristics

All studies included in this review were published in English. Nine publications from developed countries in North America and Europe accounted for 81.8% of the studies included in the analysis (Table 2). The Avanti RTVue XR AngioVue system (Optovue, Inc.) using the split-spectrum amplitude decorrelation angiography (SSADA) algorithm were the main device model and software used in studies published between 2014 and 2020 and indexed in the PubMed and Embase databases (n=6, 54.5%). Regarding the type of study, five were case series studies, three were cross-sectional studies, and three were cohort studies, one of which was a multicenter study. The mean age of the patients ranged from 58.5 to 79.7 years. The sample size of the studies ranged from 15 to 100 patients (mean=60.5 (±29.5); median=58), and the number of eyes included in the selected studies ranged from 19 to 156 (mean=66.0 (±34.6); median=72).

**Table 2:** Characteristics of the studies (authors, type of study, year of publication, country of origin, model of optical coherence tomography angiography equipment, number of eyes analyzed, and mean age of patients) included in this systematic review

Authors	Type of study	Year	Country	Model of OCTA equipment (manufacturer, country)	n (total number of patients)	Mean age±SD, years
Ahmed et al.^(16)^	Observational case series study (retrospective)	2018	Austria	OCTA SS Triton DRI (Topcon, Tokyo, Japan)	106 (98)	75.3±9.17
Carnevali et al.^(17)^	Observational case series study (prospective)	2016	France and Italy	OCT CIRRUS HD AngioPlex - model 5000 (Carl Zeiss Meditec, Inc, Dublin, CA, USA) and Avanti RTVue XR system AngioVue (Optovue, Fremont, CA, USA)	22 (20)	76.5±6.9
Corvi et al.^(18)^	Observational case series study (prospective)	2021	Italy	OCTA SS Plex Elite 9000 (Carl Zeiss Meditec, Dublin, CA, USA)	42 (42)	79.7 ±5.0
de Carlo et al.^(19)^	Observational case series study (retrospective)	2015	England	Avanti RTVue XR system AngioVue (Optovue, Inc, Fremont, CA, USA)	31 (43)	69.0 (*)
Faridi et al.^(20)^	Observational case series study (prospective)	2017	USA	Avanti RTVue XR system AngioVue (Optovue, Inc, Fremont, CA, USA)	72 (74)	76.7±8.9
Gong et al.^(21)^	Observational case series study (retrospective)	2016	China	Avanti RTVue XR system AngioVue (Optovue Inc, Fremont, CA, USA)	86 (53)	67.0 (*)
Inoue et al.^(22)^	Retrospective and multicenter observational cohort study	2016	USA and France	Avanti RTVue XR system AngioVue (Optovue, Inc, Fremont, CA, USA)	115 (100)	73.1±11.7
Moult et al.^(23)^	Observational study (transverse and prospective)	2014	USA and England	Prototype system SS-OCT (MIT, USA)	19 (15)	79.7±8.3
Nikolopoulou et al.^(24)^	Observational study (transverse)	2018	Italy	Avanti RTVue XR system AngioVue (Optovue, Inc, Fremont, CA, USA)	50 (70)	70.9±10.3
Soomro et al.^(25)^	Observational cohort study (retrospective)	2018	United Kingdom	Heidelberg Spectralis OCT2 Angiography Beta Module (Heidelberg Engineering, Heidelberg, Germany)	93 (93)	76.4±12.1
Usman et al.^(26)^	Observational study (transverse)	2019	Pakistan	Nidek RS-3000 (Nidek Inc., Gamagori, Japan)	90 (58)	58.5±5.1

* Data not available.

SD: standard deviation; MIT: Massachusetts Institute of Technology; SS: swept source; OCTA: optical coherence tomography angiography.

### Synthesis of diagnostic measures

The assessment of the diagnostic performance of OCTA revealed sensitivities above 70% in nine (81.8%) of the included studies, and the specificities exceeded 80% in seven (87.5%) of them. The sensitivity ranged from 50% to 94%, and the specificity ranged from 67.6% to 100%. Table 3 shows the sensitivity and specificity values for the diagnosis of CNV in AMD, the macular scanning pattern of OCTA, and the types of CNV evaluated.

**Table 3:** Optical coherence tomography angiography macular scanning area, sensitivity, specificity, and type of choroidal neovascularization evaluated in the included studies for the assessment of diagnostic performance

Authors	OCTA macular scanning area	Sensitivity %	Specificity %	CNV type
Ahmed et al.^(16)^	4.5mm×4.5mm 6.0mm×6.0mm	75.7	N/A	CNV Type I, CNV Type II, CNV Type III, and mixed-type CNV
Carnevali et al.^(17)^	3.0mm×3.0mm	81.8	100.0	CNV Type I
Corvi et al.^(18)^	3.0mm×3.0mm 6.0mm×6.0mm	85.7	57.1	CNV Type I, CNV Type II, CNV Type III
de Carlo et al.^(19)^	3.0mm×3.0mm 6.0mm×6.0mm	50.0	91.0	CNV Type I, CNV Type II, CNV Type III
Faridi et al.^(20)^	3.0mm×3.0mm	81.3	94.0	CNV Type I
Gong et al.^(21)^	3.0mm×3.0mm 6.0mm×6.0mm	86.5	67.6	CNV Type I, CNV Type II, CNV Type III
Inoue et al.^(22)^	3.0mm×3.0mm	66.7	N/A	CNV Type I
Moult et al.^(23)^	3.0mm×3.0mm 6.0mm×6.0mm	94.0	N/A	CNV Type I, CNV Type II
Nikolopoulou et al.^(24)^	3.0mm×3.0mm	88.0	90.0	CNV Type I, CNV Type II, CNV Type III, and mixed-type CNV
Soomro et al.^(25)^	4.3mm×2.9mm	71.0	81.0	CNV Type I, CNV Type II, CNV Type III
Usman et al.^(26)^	3.0mm×3.0mm 6.0mm×6.0mm	92.85	80.0	CNV Type I, CNV Type II

N/A: data not available; CNV: choroidal neovascularization; OCTA: optical coherence tomography angiography.

### Methodological quality and risk of bias

**Figure 2:**
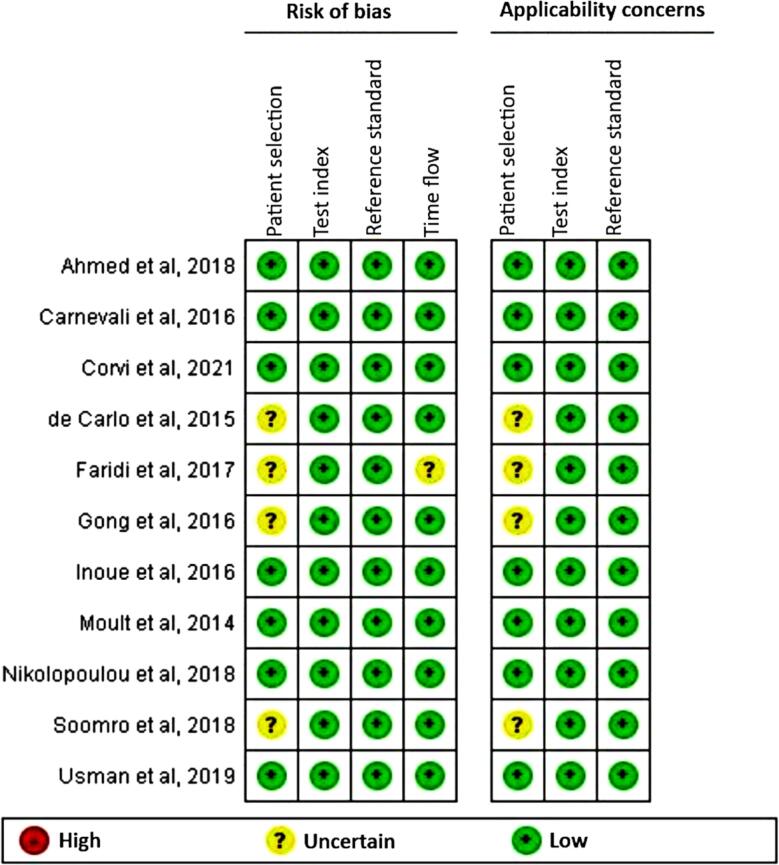
Results of the methodological quality assessment using the QUADAS-2 tool (QUADAS- Quality Assessment of Diagnostic Accuracy Studies)

Table 4 presents the results of the evaluation of the included studies across the following four domains: (1) patient selection; (2) index test; (3) reference standard; and (4) patient flow and timing. The applicability was assessed for the first three QUADAS domains. The risk of bias was determined to be low for the 11 articles included in this systematic review, indicating good methodological quality. In four studies (36.4%), the risk of bias for the patient selection domain was unclear owing to insufficient data regarding the eligibility criteria, the presence of comorbidities, or the presence of other diseases that could influence the results of the OCTA examination. In one study (9.1%), the risk bias for the patient flow and timing domain was unclear owing to a lack of information regarding the time interval between testing. Figure 2 shows a color graphic illustrating the criteria related to the assessment of the quality of the data using the QUADAS-2 tool.

**Table 4:** Assessment of the diagnostic performance of the included studies using the QUADAS-2 tool

Authors	Risk of bias (domains)				Applicability concerns		
	Patient selection	Test index	Reference standard	Patient flow and timing	Patient selection	Test index	Reference standard
Ahmed et al.^(16)^	Yes	Yes	Yes	Yes	Low	Low	Low
Carnevali et al.^(17)^	Yes	Yes	Yes	Yes	Low	Low	Low
Corvi et al.^(18)^	Yes	Yes	Yes	Yes	Low	Low	Low
de Carlo et al.^(19)^	Unclear	Yes	Yes	Yes	Unclear	Low	Low
Faridi et al.^(20)^	Unclear	Yes	Yes	Unclear	Unclear	Low	Low
Gong et al.^(21)^	Unclear	Yes	Yes	Yes	Unclear	Low	Low
Inoue et al.^(22)^	Yes	Yes	Yes	Yes	Low	Low	Low
Moult et al.^(23)^	Yes	Yes	Yes	Yes	Low	Low	Low
Nikolopoulou et al.^(24)^	Yes	Yes	Yes	Yes	Low	Low	Low
Soomro et al.^(25)^	Unclear	Yes	Yes	Yes	Unclear	Low	Low
Usman et al.^(26)^	Yes	Yes	Yes	Yes	Low	Low	Low

QUADAS: Quality Assessment of Diagnostic Accuracy Studies.

## DISCUSSION

Fundus fluorescein angiography is the current gold standard for the detection of abnormalities in the retinal vascular circulation. Choroidal neovascularization is typically identified through leakage in FFA; however, detection may be limited owing to the deficient perfusion or non-perfusion in capillaries that occurs in later phases of FFA compared to that in indocyanine green (ICG) angiography, potentially delaying the initiation of therapeutic interventions for AMD because of the reduced sensitivity for CNV detection (false negatives).^(27)^

While traditional optical coherence tomography (OCT) is a complementary approach for studying morphological disturbances of the retina, OCTA has emerged as a promising diagnostic modality for macular diseases, as it enables visualization of the retinal and choroidal vasculature and improves AMD management. Prior to the detection of exudation based on dynamic FFA information, OCTA, a non-invasive imaging technique, can enable the identification of subclinical vision-threatening lesions.^(28)^ Some of the disadvantages of FFA, including the relatively long acquisition time, the need for patient collaboration, and the possibility for adverse reactions favor the feasibility of OCTA.

Ahmed et al. reported limitations of OCTA in detecting all types of CNV lesions, specifically those of Type I, in treatment-naïve AMD cases, and priority was given to FFA as the gold standard.^(16)^ Thus, OCTA may not be effective as the sole means of diagnosing CNV, and joint interpretation of multimodal imaging involving OCT, fundus photography, FFA, and ICG angiography may be necessary to detect and monitor exudative disease activity and structural features of the retina.^(29)^

Optical coherence tomography angiography exhibited good performance in the diagnosis of CNV based on the high sensitivities found in this systematic review; however, the specificity of the technique for the diagnosis of CNV in patients with AMD may be limited owing to the fact that it is remains difficult to interpret CNV findings, and knowledge of the underlying pathophysiology and the related nomenclature are still being developed for the diagnosis of CNV. De Carlo et al. reported unsatisfactory sensitivity and argued that the finding may have correlated with the OCTA protocol applied, the small sample size, and difficulties in evaluating the images they obtained.^(19)^ Nevertheless, OCTA contributed to the initial diagnosis and facilitated improved follow-up and noninvasive monitoring of CNV. Furthermore, using OCTA, Gong et al. reported a positive predictive value of 80.4% and negative predictive value of 76.7%.^(21)^

Carnevali et al. mentioned that a minor limiting factor was trying to identify the type of CNV based on the visibility of the vascular network and the low flow rate in quiescent CNV or poor-quality images.^(17)^ Other important limitations included eye movement and projection artifacts. Gong et al. reported that a reduction in image quality was caused by poor fixation and motion artifacts arising from eye movements during image capture.^(21)^

According to Inoue et al., the main disadvantage of OCTA remains the detection of leakage secondary to CNV in type I cases, which is a visible sign in FFA assessments.^(22)^ Although it is not possible to investigate the permeability changes and apparent leakage found in FFA, OCTA can identify lesions in retinal structures that could be masked during leakage and help prevent misdiagnosis.^(22)^ In the study conducted by Soomro et al., the noted limitations of OCTA included the learning curve for grading images and interpreting the findings among retina specialists, the version of software used in combination with an experimental device, and the limited area evaluated (4.3mm×2.9mm, 15°×10°).^(25)^

Some of the potential limitations of this systematic review include the fact that it only included case series or cohort studies with small samples of AMD cases involving CNV, the absence of a control group in the design of the studies, and a possible selection bias related to the severity of the types of CNV evaluated. A recent meta-analysis conducted by Wang et al., which included nine studies, argued that differences in the etiologies of CNV, sample sizes, the maturity of diagnostic techniques, and the algorithms employed for imaging could explain the significant differences in sensitivity and specificity found in previous studies.^(30)^ However, the meta-analysis by Wang et al. reported both high sensitivity (0.83 [95% confidence interval (95%CI)=0.75–0.88]) and high specificity (0.89 [95%CI=0.79–0.94]) for detecting active CNV based on pooled values, and a higher sensitivity was noted for the nAMD group (0.88 [0.84–0.93]) compared to that for other disease groups.^(30)^

Despite the increasing use of OCTA, it is not used interchangeably with FFA owing its current limitations and the relatively lower number of scientific studies that have confirmed its effectiveness in clinical practice. ICG angiography is a valuable tool for studying choroidal circulation and detecting CNV in cases in which the use of FFA is limited due to the occurrence of subretinal or intraretinal hemorrhage; however clinical availability is restricted, and it is not broadly used in all cases.^(31,32)^ Optical coherence tomography angiography exhibited good diagnostic performance in the identification of type I and type II CNV; however, its ability to evaluate type I was more limited owing to signal attenuation below the retinal pigment epithelium (RPE) compared to that of type II CNV, and detection of type III is limited by both the positioning of the deep retinal vascular plexus towards the RPE and restricted visibility, such as in cases involving intraretinal hemorrhage or pigment epithelial detachments (PEDs).^(25,33)^ A smaller OCTA scanning pattern (3×3mm) provides better resolution and visualization of vascular network details, whereas larger sizes (6×6 and 8×8mm) in extensive CNV lesions reduce both the sensitivity of the technology itself as well as the density of scans, leading to a loss of details and increased false positive rates.^(34,35)^

Thus, it is necessary to conduct future studies to confirm the diagnostic performance of OCTA for different types of CNV in AMD and based on different types of devices and device algorithms.

## CONCLUSION

Optical coherence tomography angiography has certain advantages over other techniques, as it does not require intravenous contrast and has a fast acquisition time. The good diagnostic accuracy exhibited in the studies analyzed in this review supported its clinical utility in detecting choroidal neovascularization in age-related macular degeneration, predominantly in asymptomatic patients, and quantitative measures of choroidal neovascularization can be helpful in the monitoring of responses to follow-up treatment or exudation. Thus, we concluded that the articles published on the diagnosis of choroidal neovascularization in age-related macular degeneration using optical coherence tomography angiography exhibited high diagnostic performance, good methodological quality, and a low risk of bias in the domains evaluated using the QUADAS-2 tool.
