# Supplementary material for: Optical coherence tomography angiography for the diagnosis of choroidal neovascularization in age-related macular degeneration: a systematic review
Source: Einstein (Sao Paulo). 2025 Nov 7;23:eRW1521. doi: 10.31744/einstein_journal/2025RW1521 (PMC12671618; doi:10.31744/einstein_journal/2025RW1521)
Supplement: Supplementary file 1 [file 2317-6385-eins-23-eRW1521-suppl1.pdf]

SUPPLEMENTARY MATERIAL

Optical coherence tomography angiography for the diagnosis of choroidal neovascularization in age-related macular degeneration: a systematic review

Tarciana de Souza Soares, Amanda dos Santos Cristino, Analmiria de França Silva, Leticia Ribeiro dos Santos

DOI: 10.31744/einstein_journal/2025RW1521

Tabel 1S. Search strategy

Database	Descriptors	Search query
PubMed/MED LINE	Mesh	(Optical Coherence Tomography Angiography OR Optical Coherence Tomography Angiogram OR OCTA OR OCT-A OR OCT-Angiography OR OCT Angiography) AND (Age-related macular degeneration OR Maculopathy OR AMD OR Wet Macular Degeneration OR Retinal Degeneration OR Choroidal neovascularization OR Choroidal Neovascularizations OR Choroid Neovascularization OR Choroid Neovascularizations) AND (Fluorescein angiography OR Fluorescence Angiography OR Fundus Fluorescence Photography OR FFA) AND (Diagnose OR Diagnosis OR Diagnostic imaging) NOT (Animal Research OR Animal Experimental Use OR Animal Experimental Uses OR Animal Experiments OR Animal Experiment) NOT (Case Reports OR Case Study OR Case Studies OR Case Histories) NOT (In Vitro Technique OR In Vitro Testing OR In Vitro Testings OR In Vitro Tests OR In Vitro Test)
EMBASE	Emtree	(Optical Coherence Tomography Angiography OR swept source optical coherence tomography angiography OR optical coherence tomography angiography device) AND (age related macular degeneration OR wet macular degeneration OR macular degeneration OR Retina Degeneration OR neovascularization) AND (fluorescence angiography OR retina fluorescein angiography) AND (diagnosis OR Diagnostic imaging) NOT (Animal Research OR Animal Experiment) NOT (Case Report OR case control study OR Case Study) NOT (in vitro study)
Cochrane Library	Mesh	(Optical Coherence Tomography Angiography OR Optical Coherence Tomography Angiogram OR OCTA OR OCT-A OR OCTAngiography OR OCT Angiography) AND (Age-related macular degeneration OR Maculopathy OR AMD OR Wet Macular Degeneration OR Retinal Degeneration OR Choroidal neovascularization OR Choroidal Neovascularizations OR Choroid Neovascularization OR Choroid Neovascularizations) AND (Fluorescein angiography OR Fluorescence Angiography OR Fundus Fluorescence Photography OR FFA) AND (Diagnose OR Diagnosis OR Diagnostic imaging) NOT (Animal Research OR Animal Experimental Use OR Animal Experimental Uses OR Animal Experiments OR Animal Experiment) NOT (Case Reports OR Case Study OR Case Studies OR Case Histories) NOT (In Vitro Technique OR In Vitro Testing OR In Vitro Testings OR In Vitro Tests OR In Vitro Test)
Scielo	Decs/ Mesh	(Degeneração macular relacionada à idade OU Maculopatia Relacionada à Idade OU Degeneração macular exsudativa OU Degeneração Macular Hemorrágica OU Degeneração Macular Úmida OU Neovascularização de coróide OU Neovascularização Coroidal OU Neovascularização Coroideana) E (Tomografia de Coerência Óptica OU Angiografia OU Angiofluoresceinografia OU Angiografia com Fluoresceína OU Angiografia de Fluorescência OU Fotografia de Fluorescência de Fundo) E (Diagnóstico OU Diagnóstico por Imagem) NÃO (Experimentos em animais) NÃO (Relatos de caso OU Apresentação de Caso OU Apresentação de Casos OU Estudo de Caso OU Estudo de Casos OU Histórico de Caso OU Histórico de Casos OU Histórico do Caso OU Histórico dos Casos OU Históricos dos Casos) NÃO (In Vitro como Assunto OU Método In Vitro OU Técnica In Vitro OU Testagem In Vitro OU Teste In Vitro OU Testes In Vitro)
Lilacs	Mesh	(Optical Coherence Tomography Angiography OR Optical Coherence Tomography Angiogram OR OCTA OR OCT-A OR OCTAngiography OR OCT Angiography) AND (Age-related macular degeneration OR Maculopathy OR AMD OR Wet Macular Degeneration OR Retinal Degeneration OR Choroidal neovascularization OR Choroidal Neovascularizations OR Choroid Neovascularization OR Choroid Neovascularizations) AND (Fluorescein angiography OR Fluorescence Angiography OR Fundus Fluorescence Photography OR FFA) AND (Diagnose OR Diagnosis OR Diagnostic imaging) NOT (Animal Research OR Animal Experimental Use OR Animal Experimental Uses OR Animal Experiments OR Animal Experiment) NOT (Case Reports OR Case Study OR Case Studies OR Case Histories) NOT (In Vitro Technique OR In Vitro Testing OR In Vitro Testings OR In Vitro Tests OR In Vitro Test)
Periódico da Capes	Decs	(Degeneração macular relacionada à idade OU Maculopatia Relacionada à Idade OU Degeneração macular exsudativa OU Degeneração Macular Hemorrágica OU Degeneração Macular Úmida OU Neovascularização de coróide OU Neovascularização Coroidal OU Neovascularização Coroideana) E (Tomografia de Coerência Óptica OU Angiografia OU Angiofluoresceinografia OU Angiografia com Fluoresceína OU Angiografia de Fluorescência OU Fotografia de Fluorescência de Fundo) E (Diagnóstico OU Diagnóstico por Imagem) NÃO (Experimentos em animais) NÃO (Relatos de caso OU Apresentação de Caso OU Apresentação de Casos OU Estudo de Caso OU Estudo de Casos OU Histórico de Caso OU Histórico de Casos OU Histórico do Caso OU Histórico dos Casos OU Históricos dos Casos) NÃO (In Vitro como Assunto OU Método In Vitro OU Técnica In Vitro OU Testagem In Vitro OU Teste In Vitro OU Testes In Vitro)
